# Hepatic GHR deletion alters bile acid composition and links reduced FXR signaling to hepatic steatosis

**DOI:** 10.3389/fendo.2026.1919362

**Published:** 2026-10-15

**Authors:** Liyuan Ran, Ruolan Li, Yu Wang, Xinyu Liu, Yingjie Wu, Zichao Yu

**Affiliations:** 1 Metabolism and Disease Research Center, Central Hospital Affiliated to Shandong First Medical University, Jinan, China; 2 School of Laboratory Animal and Shandong Laboratory Animal Center, Shandong First Medical University, Jinan, Shandong, China; 3 Key Laboratory of Endocrine Glucose & Lipids Metabolism and Brain Aging, Ministry of Education, Shandong Provincial Hospital Affiliated to Shandong First Medical University, Jinan, Shandong, China

**Keywords:** bile acid, FXR, growth hormone, growth hormone receptor, MASLD

## Abstract

**Aims:**

Metabolic dysfunction-associated steatotic liver disease (MASLD) is a growing global health concern. Growth hormone (GH) signaling regulates hepatic lipid metabolism, but its relationship with the bile acid–farnesoid X receptor (FXR) axis in MASLD remains unclear. We investigated the effects of hepatic GH receptor (GHR) deletion on bile acid metabolism and hepatic steatosis and evaluated the response to pharmacological FXR activation.

**Materials and methods:**

Liver-specific *Ghr* knockout (LKO) mice and LL controls were examined using targeted metabolomics, bile acid profiling, biochemical assays, histology, and gene and protein expression analyses. Public human MASLD transcriptomic datasets were analyzed for GHR/IGF1–FXR expression relationships. The effects of the FXR agonist GW4064 were evaluated in LKO mice and primary LKO hepatocytes.

**Results:**

Hepatic GHR deletion promoted steatosis, increased hepatic total and conjugated bile acids, including TαMCA, TβMCA, and TUDCA, and reduced BSEP and FXR/SHP expression. Gallbladder total and conjugated bile acids were lower in LKO mice. Hepatic GHR or IGF1 expression correlated positively with FXR expression in human MASLD datasets. GW4064 ameliorated steatosis and reduced serum markers of liver injury in LKO mice and decreased lipid accumulation in primary LKO hepatocytes. Treatment increased lipolysis-associated gene expression without significantly changing the measured lipogenic genes or *Bsep* expression.

**Conclusions:**

Hepatic GHR deletion disrupts bile acid homeostasis and promotes steatosis. GW4064 improves steatosis in this model, supporting FXR activation as a potential intervention for GHR-deficient steatosis. The altered bile acid pool may contribute to reduced hepatic FXR signaling.

## Introduction

Bile acids, once considered merely as detergents for lipid digestion, are now recognized as crucial signaling metabolites that govern systemic metabolic homeostasis. Synthesized in hepatocytes through the classic pathway (yielding mainly cholic acid, CA) and alternative pathway (yielding mainly chenodeoxycholic acid, CDCA and its derivatives), primary bile acids are conjugated with glycine and taurine before being exported to the intestine, where they undergo microbial modification into secondary bile acids (1). Bile acids regulate lipid metabolism primarily by interacting with bile acid receptors, notably the farnesoid X receptor (FXR) and the G protein-coupled receptor 5 (TGR5). The bile acid–FXR axis plays a pivotal role in hepatic lipid metabolism by suppressing triglyceride synthesis and promoting lipid clearance (2, 3). Consequently, dysregulation of bile acid homeostasis and impaired FXR signaling are strongly implicated in the pathogenesis of metabolic diseases, including metabolic dysfunction-associated steatotic liver disease (MASLD) (4, 5).

The pituitary-secreted growth hormone (GH) plays a pivotal role in regulating diverse physiological processes, including growth and development, metabolism, immunity, and ageing (6–8). Targeting various organs such as the liver, adipose tissue, and muscle, GH exerts its effects by binding to its membrane receptor (GHR). Activated GHR initiates intracellular signaling cascades and stimulates insulin-like growth factor 1 (IGF1) production, mediating pleiotropic effects synergistically with or independently of IGF1 (7). Consistently, dysregulated GH signaling has been implicated in the pathogenesis of several diseases, including obesity, insulin-resistant diabetes, and cancer (9, 10). Accumulating evidence has highlighted a previously underrecognized association between impaired GH signaling and MASLD. Circulating GH levels have been observed to decline in both pediatric and adult MASLD patients (11–13). Similarly, adults with Laron syndrome, caused by inactivating GHR mutations, exhibit a high prevalence of MASLD (14). Mouse models with liver-specific deletion of key molecules within the GH signaling pathway, such as GHR and JAK2/STAT5, consistently develop hepatic steatosis or steatohepatitis, underscoring the direct role of hepatocyte GH signaling in lipid homeostasis (15–17). While GH is known to regulate lipid metabolism by inhibiting *de novo* lipogenesis and inducing white adipose tissue lipolysis (18–20), the underlying mechanisms of GH regulation of hepatic lipid metabolism are still not fully understood. Intriguingly, emerging evidence suggests a potential intersection between GH signaling and bile acid metabolism, as both GH administration and genetic disruption of the GH pathway can modulate the expression of bile acid transporters (21–23). However, how GH signaling regulates the bile acid–FXR axis, and whether this relationship contributes to MASLD pathogenesis, has not been defined.

MASLD is a burgeoning public health concern associated with increasing morbidity, even among younger individuals (24–26). Characterized by excessive hepatic fat accumulation, MASLD encompasses a pathological spectrum ranging from simple steatosis (or nonalcoholic fatty liver, NAFL) to nonalcoholic steatohepatitis (NASH). Its complex etiology, as described by the “multiple parallel hits” hypothesis, involves intricate crosstalk among endocrine, metabolic, and gut-derived factors (27). Within this framework, both impaired bile acid–FXR signaling and deficient GH action constitute significant pathogenic “hits”, yet their potential mechanistic interplay has not been investigated.

In this study, we investigated the effects of hepatic GHR deletion on bile acid composition, hepatic FXR signaling, and steatosis, and evaluated the metabolic response to pharmacological FXR activation. We combined analyses of liver-specific *Ghr* knockout mice and primary hepatocytes with public human MASLD transcriptomic datasets to examine the relationship between hepatic GH signaling and FXR. Our findings show that hepatic GHR regulates bile acid and lipid homeostasis and that FXR activation may improve steatosis caused by hepatic GHR deficiency.

## Materials and methods

### Animal studies and sample collection

The hepatic tissue-specific *Ghr* knockout (LKO) mice used in this study were generated by crossing GHR^flox/flox^ (LL) mice (28, 29) with Albumin-Cre mice (B6.FVB(129)-Tg(Alb1-cre)1Dlr/J, Jackson Laboratory, Stock No. 016832). LL mice served as controls. Mice were maintained on regular chow (1010009, 12.79% kcal from fat, Xietong Bioengineering Company, Nanjing, China) and housed in an individually ventilated cage (IVC) system with a 12-hr/12-hr dark/light cycle, with free access to food and water. Mice were fasted overnight before terminal blood and tissue collection. Male LL and LKO mice were anesthetized via intraperitoneal injection of Avertin (20 μL/g) and subsequently euthanized by exsanguination at 24 weeks of age. To study the effects of FXR, 22-week-old male LKO mice were treated with FXR agonist GW4064 (M2018, AbMole, TX, USA) by intraperitoneal injection once daily at 30 mg/kg body weight for two weeks, and the control group was treated with vehicle. The dose, route, frequency, and duration followed previously reported murine GW4064 regimens (30, 31). Sample sizes for individual assays are specified in the figure legends. Animal care and experimental procedures adhered to the established guidelines for laboratory animal treatment and were approved by the Committee on the Ethics of Animal Experiments of Shandong Provincial Hospital Affiliated to Shandong First Medical University & Shandong Academy of Medical Sciences.

Liver and gallbladder weights were recorded, with a portion of the liver fixed in 10% formalin for histological analysis, and the remainder snap-frozen in liquid nitrogen. Blood samples were collected by retro-orbital bleeding, and serum was isolated by centrifugation at 1000 g for 10 min at 4 °C. All samples were stored at −80 °C for subsequent use.

### Primary hepatocyte isolation and GW4064 treatment

Primary hepatocytes were isolated from 24-week-old male LKO mice. Briefly, mice were anesthetized with Avertin. After portal-vein cannulation, the liver was perfused for 5 min with Hanks’ balanced salt solution (C0218, Beyotime, Shanghai, China) containing 0.5 mM EDTA and 10 mM HEPES, followed by perfusion for 6 min with a type IV collagenase (C4–28, Sigma, MO, USA) solution (50 mg collagenase per 100 mL DMEM supplemented with 5 mM CaCl_2_ and 10 mM HEPES). The digested liver was excised and gently teased apart in ice-cold DMEM containing 5% fetal bovine serum (FBS). The resulting cell suspension was filtered through a 70-μm nylon strainer. Hepatocytes were pelleted by centrifugation at 50 × g for 2 min and washed twice with culture medium. Viability was assessed by Trypan blue exclusion, and only preparations with >85% viable cells were used. Cells were plated on collagen-coated dishes in DMEM medium with 10% FBS, 100 U/ml penicillin, 100 μg/ml streptomycin, 2 mM L-glutamine, 1 μM dexamethasone, and 4 μg/ml insulin. After 4 h, the medium was replaced with fresh culture medium to remove unattached cells. Following overnight adaptation in serum-free medium, hepatocytes were treated with 1 μM GW4064 (HY-50108, MedChemExpress, Shanghai, China) or vehicle containing 0.1% dimethyl sulfoxide (DMSO) for 24 h (32) before RNA extraction or Oil Red O staining.

### Biochemical parameter analysis

The hepatic triglyceride (TG) content was measured with the Triglyceride assay kit (A110-1, Jian Cheng Biological Engineering Institute, Nanjing, China) according to the manufacturer’s instructions. The activities of aspartate aminotransferase (AST), alanine aminotransferase (ALT) and alkaline phosphatase (ALP) as well as the levels of total cholesterol (TC), low-density lipoprotein (LDL-C), and triglyceride in serum were assessed with a biochemical analyzer (BS-830, Mindray Bio-Medical, Shenzhen, China).

### Histological analysis

Liver samples fixed in 10% formalin were dehydrated, embedded in paraffin, and then sliced into 8 μm sections, which were subsequently stained with hematoxylin-eosin (H&E, SI106-02, Sevenbio, Beijing, China). For Oil Red-O staining, liver samples were sucrose dehydrated, cut into 10 µm sections at -20 °C, and stained with Oil Red-O (D027, Jian Cheng Biological Engineering Institute). Digital images were acquired with an Axio Observer microscope (ZEISS, Oberkochen, Germany).

Intracellular lipid accumulation in primary hepatocytes was assessed by Oil Red O staining. After treatments, hepatocytes cultured in six-well plates were washed twice with ice-cold phosphate-buffered saline (PBS) and fixed with 4% paraformaldehyde for 30 min at room temperature. Cells were washed twice with PBS, rinsed with 60% isopropanol for 2 min, and stained with Oil Red O working solution for 30 min at room temperature in the dark. Excess stain was removed with 60% isopropanol, followed by three washes with distilled water. Lipid droplets were imaged with an inverted light microscope at 10× magnification. Five fields were analyzed per group. Oil Red O-positive area was quantified using ImageJ and expressed as a percentage of the estimated cell-covered area.

### Targeted metabolomics and bile acids analysis

Targeted quantitative metabolomics analysis was performed by Metabo-Profile (Shanghai, China). The Q300™ primary targeted metabolic profiling platform, based on a triple quadrupole mass spectrometer coupled with ultrahigh performance liquid chromatography (UPLC-QQQ-MS), was used to evaluate the metabolic profile of liver samples. This platform enables the identification of ~300 targeted metabolites, including amino acids, fatty acids, bile acids, organic acids, carbohydrates, lipids, and more. The bile acid profile in both liver and gallbladder was determined by ultrahigh performance liquid chromatography-tandem mass spectrometry (UPLC-MS/MS) with the BAP Ultra kit (Metabo-Profile, Shanghai, China).

### Weighted gene co-expression network analysis

Weighted gene co-expression network analysis (WGCNA) was conducted using the WGCNA package in R software (33) to identify metabolite modules associated with MASLD-related phenotypes. The analysis used 214 metabolites from 18 liver samples (six LL, six LKO, and six adipose-tissue-specific *Ghr* knockout [AKO] mice) from our previously published study of tissue-specific GHR disruption (22). All three genotype groups from that dataset were included in network construction and module–trait analysis. A Pearson correlation network was constructed using a soft-thresholding power of 10. Modules were identified by hierarchical clustering of topological overlap-based dissimilarities, with a minimum module size of 10, deepSplit = 2, and a module merging cut height of 0.25. Module eigengenes (MEs) were correlated with MASLD-related phenotypes using Pearson correlation, and the blue module was selected for further characterization. Module membership (MM) and biomarker significance (BS) were defined as the correlations of each metabolite profile with its ME and the corresponding phenotype, respectively. Module stability was assessed using 1,000 genotype-stratified bootstrap resamplings adapted from the SABRE framework (34). Six samples were drawn with replacement within each genotype group, and networks were reconstructed using the same parameters. Each reference module was independently matched to the reconstructed non-grey module with the highest modified overlap similarity, calculated as the number of shared metabolites divided by the size of the smaller module; standard Jaccard similarity resolved ties. Stability was summarized using the SABRE H-index, defined as the largest value h for which at least a proportion h of resamplings achieved a similarity of at least h. MEs of the reference and matched modules were recalculated within each bootstrap dataset, and their absolute Pearson correlations were determined.

### RNA extraction and real-time qPCR

RNA was extracted from liver samples or cultured primary hepatocytes using the Total RNA Extraction Kit (SM132, Sevenbio). RNA concentration was measured using a NanoDrop One spectrophotometer (Thermo Fisher, MA, USA). Subsequently, total RNA was reverse-transcribed using the 1st Strand cDNA Synthesis Kit (11139ES60, Yeasen, Shanghai, China). Target-gene expression was measured by qPCR using qPCR SYBR Green Master Mix (11202ES08, Yeasen) on the QuantStudio 7 Pro Real-Time PCR System (Applied Biosystems, CA, USA). Relative expression was calculated using the 2^−ΔΔCt^ method with *Gapdh* as the reference gene. Primer sequences used in this study are listed in **Supplementary Table S1**.

### Protein extraction and Western blot

Proteins were extracted from liver samples using RIPA lysis buffer (SW104, Sevenbio) supplemented with protease and phosphatase inhibitors (SW106 and SW107, Sevenbio). The protein concentration was determined by the BCA Protein Assay Kit (P0012, Beyotime). Proteins were separated on SDS-PAGE, transferred onto a nitrocellulose membrane, and probed with specific primary antibodies. The membrane was then incubated with an HRP-conjugated secondary antibody, and the chemiluminescent signals were detected using ECL Plus (1810202, ClinX, Shanghai, China). Antibodies used in this study were listed as follows: FXR (25055-1-AP, Proteintech, Wuhan, China), SHP (A16454, ABclonal, Wuhan, China), CYP7B1 (ABP57039, Abbkine, Wuhan, China), BSEP (PB9414, Boster, Wuhan, China), FASN (66591-1-lg, Proteintech), HSL (17333-1-AP, Proteintech), ATGL (A6245, ABclonal), ACC1 (67373-1-lg, Proteintech), SCD1 (28678-1-AP, Proteintech), GAPDH (AC002, ABclonal), β-Actin (AC026, ABclonal), β-Tubulin (AC021, ABclonal), HRP-conjugated Goat Anti-Rabbit IgG (SA00001-2, Proteintech), HRP-conjugated Goat Anti-Mouse IgG (SA00001-1, Proteintech).

### GEO data analysis

The raw gene-level count read data from the liver of two MASLD cohorts were retrieved from the GEO database (https://www.ncbi.nlm.nih.gov/geo/) under the accession number of GSE135251 (35) and GSE48452 (36). DESeq2 (37) was used to analyze gene expression levels across various groups. The correlation between different genes was evaluated using Spearman correlation coefficient analysis and visualized by ggplot2 (38) in R.

### Statistical analysis

Data are presented as mean ± SEM unless otherwise specified. Two-tailed unpaired Student’s t-tests were used for comparisons between two groups. For bile acid analyses, *P* values were adjusted using the Benjamini–Hochberg false discovery rate (FDR) method, with adjusted *P* values (q values) < 0.05 considered significant. For other two-group comparisons, *P* < 0.05 was considered significant. Exact sample sizes and significance symbols are specified in the figure legends.

## Results

### Bile acid metabolism is correlated with MASLD in liver-specific *Ghr* knockout mice

The LKO mice generated by our group exhibited MASLD-related phenotypes consistent with congenital and adult-onset liver-specific *Ghr* knockout or knockdown models (18, 39). Liver weight and the liver-to-body-weight ratio were higher in LKO mice than in LL controls (**Figures 1A, B**). H&E staining showed hepatic steatosis, and hepatic triglyceride content was markedly increased (**Figures 1C, D**). Serum AST, ALT, ALP, TC and LDL-C were also elevated, whereas serum triglycerides did not differ significantly between LL and LKO mice (**Figures 1E, F**).

**Figure 1:**
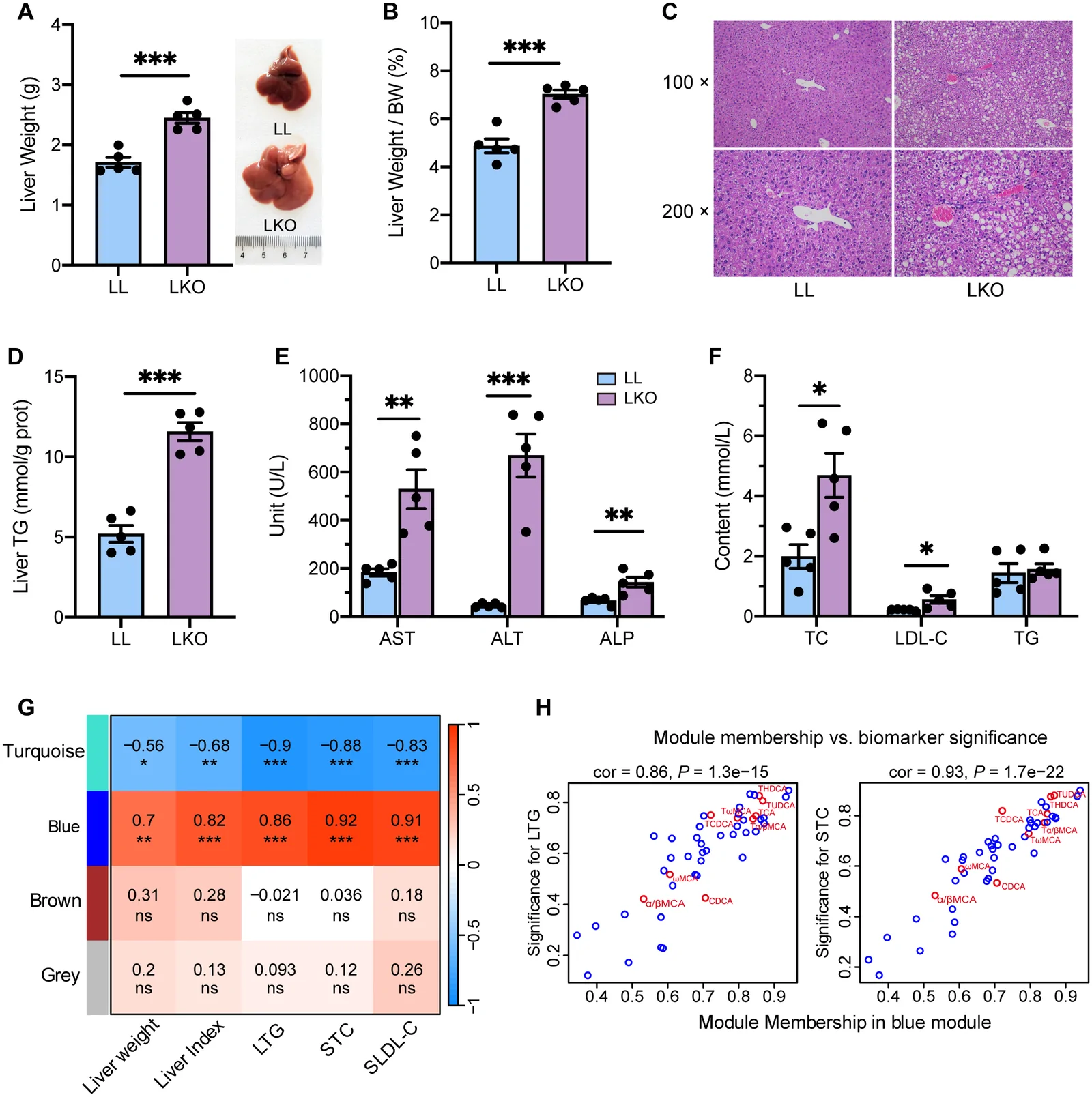
MASLD-related phenotypes and their association with hepatic metabolites in LKO mice. **(A)** Liver weight and representative gross liver images from LL and LKO mice. **(B)** Liver-to-body-weight ratio. **(C)** Representative H&E-stained liver sections. **(D)** Hepatic triglyceride content. **(E)** Serum AST, ALT, and ALP activities. **(F)** Serum total cholesterol, LDL-C, and triglyceride concentrations. **(G)** WGCNA module–trait heatmap showing Pearson correlation coefficients between module eigengenes and MASLD-related phenotypes. Correlation coefficients and significance symbols are displayed in the cells. **(H)** Relationships between module membership in the blue module and biomarker significance for hepatic triglycerides (LTG) and serum total cholesterol (STC). For **(A–F)**, data are mean ± SEM; n = 5 mice per group. *P < 0.05, **P < 0.01, and ***P < 0.001 by two-tailed unpaired Student’s t-test. For **(G)**, *P < 0.05, **P < 0.01, and ***P < 0.001 based on Pearson correlation P values; ns, not significant. Correlation statistics for **(G–H)** are shown in the corresponding panels. The WGCNA analyses shown in panels G and H were performed using metabolomics data from our previous study (22).

Targeted metabolomics assay of liver samples and WGCNA analysis were performed to investigate metabolic changes associated with MASLD onset in LKO mice. The detected metabolites were assigned to three modules (blue, brown, and turquoise), with unassigned metabolites designated grey. Across 1,000 stratified SABRE bootstrap resamplings, the blue, brown, and turquoise modules achieved H-indices of 0.640, 0.583, and 0.679, respectively (**Supplementary Figure S1A**). The corresponding median absolute ME correlations were 0.935, 0.893, and 0.968 (**Supplementary Figure S1B**), supporting the reproducibility of the principal module patterns and their eigengene profiles. The blue module showed strong positive correlations with MASLD-related indicators, including liver index, liver triglyceride (LTG), and serum total cholesterol (STC) (**Figure 1G**). Within this module, MM was significantly correlated with BS for LTG and STC (**Figure 1H**). Several bile acids, including TUDCA, TαMCA, and TβMCA, had high MM and BS values, linking bile acid metabolism to the MASLD-related phenotypes in LKO mice.

### Impaired GH pathway is associated with the downregulation of FXR

FXR is an important bile acid nuclear receptor that regulates several metabolic pathways. Hepatic *Fxr* and *Shp* mRNA and protein levels were lower in LKO mice than in LL controls (**Figures 2A–C**), indicating FXR pathway inhibition in these mice. We then examined the public human liver transcriptomic datasets of MASLD patients. In GSE48452, hepatic GHR expression was lower in samples classified as NAFL (simple fatty liver) or NASH (steatohepatitis) than in control samples, and GHR expression correlated positively with FXR expression (**Figures 2D–F**). In GSE135251, hepatic IGF1 expression, used here as an indirect downstream transcriptional readout of GHR–STAT5 signaling, was also lower in NAFL and NASH and correlated positively with FXR expression (**Figures 2G–I**). The positive GHR/IGF1–FXR correlations in the human datasets were consistent with the lower FXR/SHP expression observed in LKO liver.

**Figure 2:**
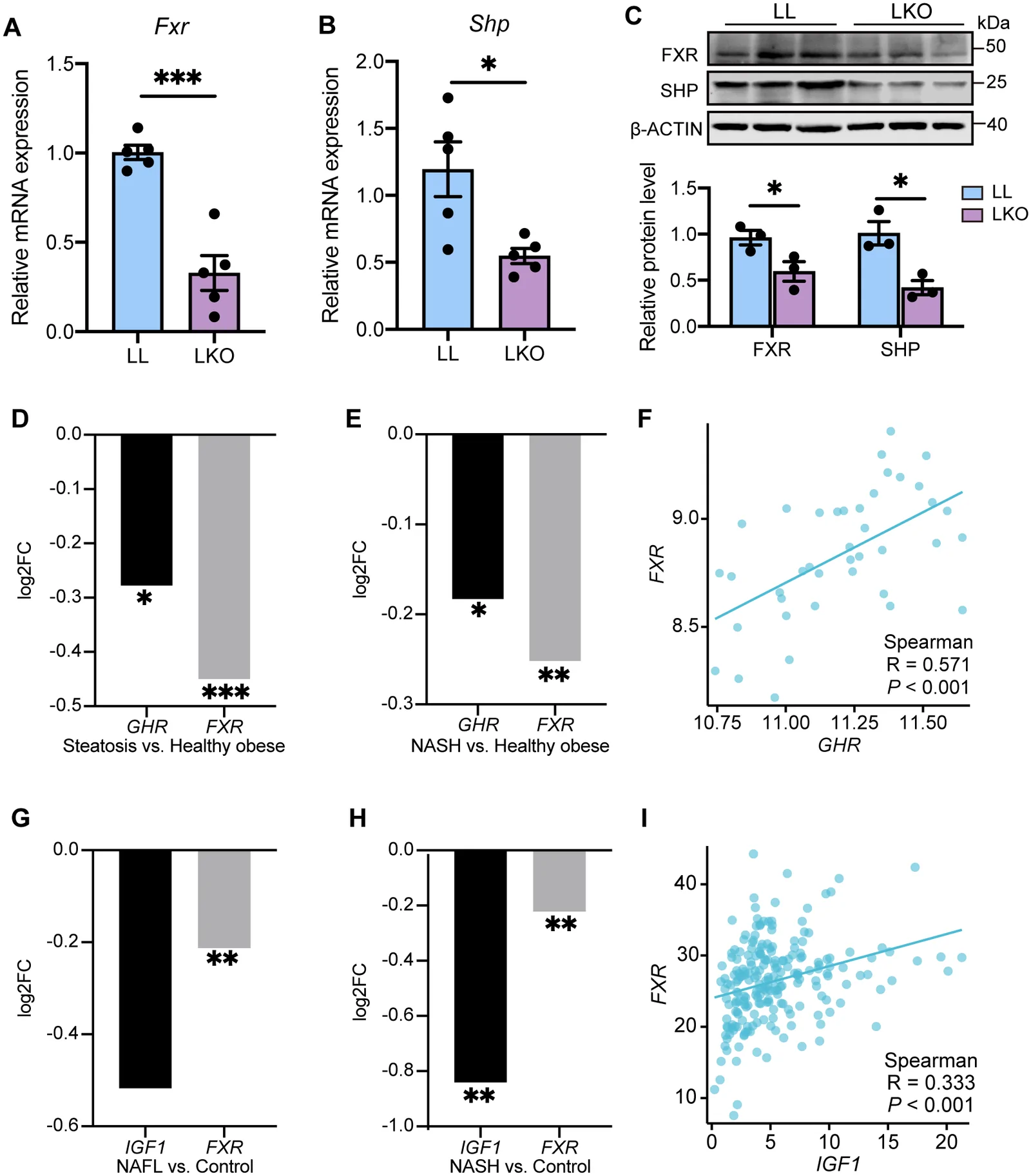
Hepatic GHR/IGF1 and FXR pathway-related expression in LKO mice and public human MASLD transcriptomic datasets. **(A, B)**. Hepatic *Fxr* **(A)** and *Shp* **(B)** mRNA expression in LL and LKO mice (n = 5 mice per group). **(C)** Representative immunoblots and quantification of hepatic FXR and SHP protein abundance in LL and LKO mice (n = 3 mice per group). **(D–F)** Hepatic GHR and FXR mRNA expression in control, NAFL, and NASH samples from GSE48452 **(D, E)** and their Spearman correlation **(F)**. **(G–I)**. Hepatic IGF1 and FXR mRNA expression in control, NAFL, and NASH samples from GSE135251 **(G, H)** and their Spearman correlation **(I)**. Data are mean ± SEM where applicable. For **(A–C)**, groups were compared using a two-tailed unpaired Student’s t-test. For **(D–I)**, differential expression was analyzed with DESeq2 and correlations with Spearman’s method. *P < 0.05, **P < 0.01, and ***P < 0.001.

### FXR-antagonistic bile acids are elevated in LKO mice

To examine whether altered bile acid composition accompanied reduced hepatic FXR/SHP expression, we profiled the hepatic bile acid pool. Total hepatic bile acids were higher in LKO mice (**Figure 3A**), largely reflecting an increase in conjugated species (**Figure 3B**), whereas unconjugated bile acids did not differ significantly (**Figure 3C**). Especially, FXR-antagonistic bile acids, including TαMCA, TβMCA, and TUDCA, were enriched in LKO liver (**Figures 3D–G**). Thus, hepatic accumulation of these conjugated bile acids accompanied lower FXR/SHP expression.

**Figure 3:**
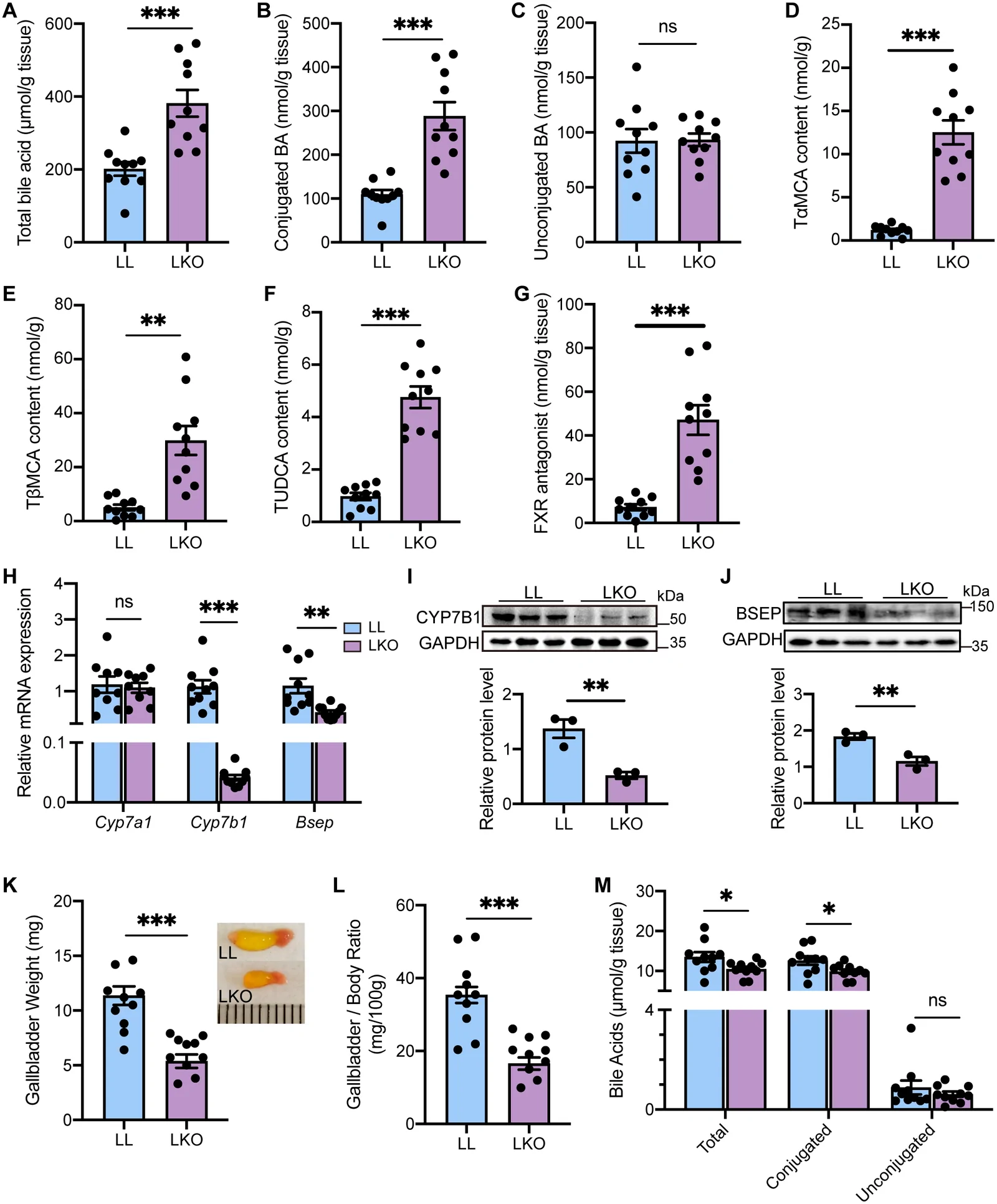
Hepatic and gallbladder bile acid profiles and bile acid-related gene expression in LL and LKO mice. **(A)** Hepatic total bile acid content. **(B)** Hepatic conjugated bile acid content. **(C)** Hepatic unconjugated bile acid content. **(D–G)**. Hepatic concentrations of FXR-antagonistic bile acids, including TαMCA, TβMCA, and TUDCA in LL and LKO mice. **(H)** Hepatic *Cyp7a1*, *Cyp7b1*, and *Bsep* mRNA expression. **(I)** Representative immunoblot and quantification of hepatic CYP7B1 protein abundance. **(J)** Representative immunoblot and quantification of hepatic BSEP protein abundance. **(K)** Gallbladder weight. **(L)** Gallbladder-to-body-weight ratio. **(M)** Gallbladder total, conjugated, and unconjugated bile acid contents. Data are mean ± SEM. Unless otherwise stated, n = 10 mice per group; n = 3 mice per group for immunoblotting in **(I–J)**. Two-tailed unpaired Student’s t-tests were used. For **(A–G, M)** *q < 0.05, **q < 0.01, and ***q < 0.001; for **(H–L)**, *P < 0.05, **P < 0.01, and ***P < 0.001.

To explore factors associated with this altered bile acid profile, we examined hepatic genes involved in bile acid synthesis and transport. *Cyp7a1*, which encodes the rate-limiting enzyme in bile acid synthesis, was unchanged (**Figure 3H**). *Cyp7b1* mRNA and CYP7B1 protein, which are involved in non-12-OH bile acid synthesis, were both lower in LKO mice (**Figures 3H,I**). We next examined the bile salt export pump gene (*Bsep*), which was significantly downregulated in LKO mice at both the mRNA and protein levels (**Figures 3H, J**). These changes coincided with lower gallbladder weight and gallbladder-to-body-weight ratio (**Figures 3K, L**), together with reduced total and conjugated bile acids in the gallbladder (**Figure 3M**). Gallbladder unconjugated bile acids did not differ significantly. Together, these findings support altered hepatobiliary bile acid compartmentalization in LKO mice. Lower BSEP expression may contribute to this redistribution of conjugated bile acids.

### Systemic GW4064 treatment ameliorates steatosis in LKO mice

We next treated LKO mice with the FXR agonist GW4064 to test whether pharmacological FXR activation could reverse the metabolic phenotype. GW4064 increased hepatic *Fxr* expression and induced *Shp* at the mRNA and protein levels (**Figures 4A,B**), confirming activation of the FXR signaling. Body weight did not differ significantly between groups (**Figure 4C**). GW4064 significantly reduced liver weight and the liver-to-body-weight ratio (**Figures 4D,E**) and improved hepatic steatosis as indicated by H&E and Oil Red O staining (**Figure 4F**). Serum AST, ALT, ALP, TC, LDL-C and TG were also reduced (**Figures 4G,H**). Notably, despite *Bsep* being a downstream target of FXR, its expression remained without a significant change following GW4064 treatment. This observation was in line with the unchanged gallbladder weight and the gallbladder-to-body-weight ratio (**Supplementary Figures S2B, C**). Thus, FXR activation improved hepatic steatosis and the associated biochemical abnormalities in LKO mice.

**Figure 4:**
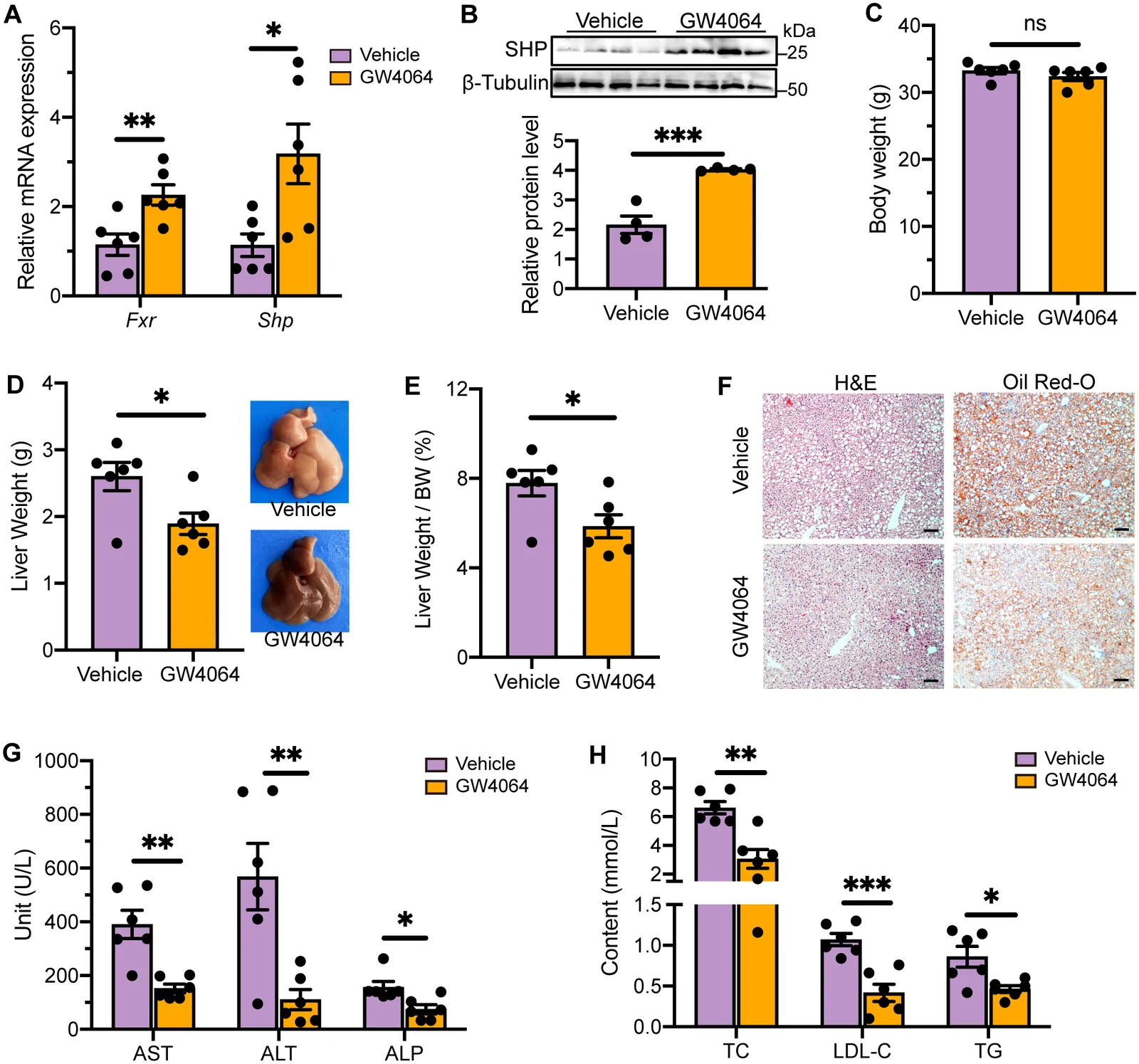
Effects of systemic GW4064 treatment on MASLD-related phenotypes in LKO mice. **(A)** Hepatic *Fxr* and *Shp* mRNA expression in vehicle- and GW4064-treated LKO mice. **(B)** Representative immunoblot and quantification of hepatic SHP protein. **(C)** Body weight. **(D)** Liver weight and representative gross liver images. **(E)** Liver-to-body-weight ratio. **(F)** Representative H&E- and Oil Red O-stained liver sections (scale bar, 100 μm). **(G)** Serum AST, ALT, and ALP activities. **(H)** Serum total cholesterol, LDL-C, and triglyceride concentrations. Data are mean ± SEM. Unless otherwise stated, n = 6 mice per group; n = 4 mice per group for immunoblotting in **(B)**. *P < 0.05, **P < 0.01, and ***P < 0.001 by two-tailed unpaired Student’s t-test.

### GW4064 selectively increases the expression of lipolysis-associated genes in LKO mice

Fine-tuned lipid homeostasis is crucial for liver health. Key enzymes involved in *de novo* lipogenesis include acetyl-CoA carboxylase 1 (ACC1), fatty acid synthase (FASN) and stearoyl-CoA desaturase-1 (SCD1). Adipose triglyceride lipase (ATGL) and hormone-sensitive lipase (HSL) mediate triglyceride hydrolysis, with ATGL catalyzing the initial rate-limiting step and HSL hydrolyzing diglycerides (**Figure 5A**). Compared with LL controls, LKO mice showed lower expression of *Atgl* and *Hsl* and higher expression of *Acc*, *Fasn*, and *Scd1* (**Figure 5B**). In GW4064-treated LKO mice, mRNA and protein levels of *Atgl* and *Hsl* were significantly increased, whereas the measured lipogenic genes and proteins were unchanged (**Figures 5C–E**). The response to GW4064 therefore centered on lipolysis-associated genes rather than reversal of the lipogenic program.

**Figure 5:**
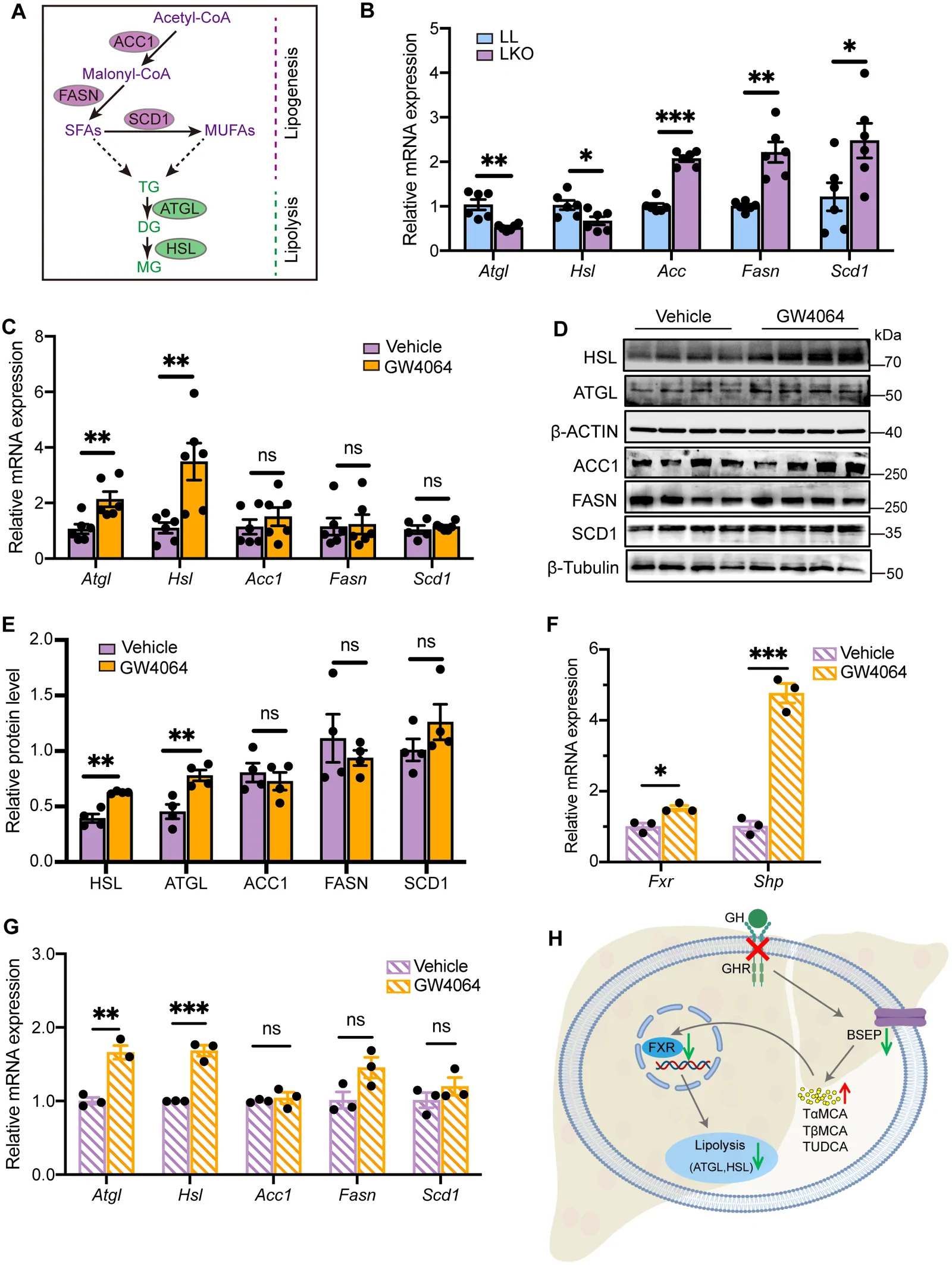
Effects of hepatic GHR deletion and GW4064 treatment on lipid metabolism-related gene and protein expression. **(A)** Schematic overview of *de novo* lipogenesis and triglyceride-hydrolysis pathways and the measured regulators. **(B)** Hepatic mRNA expression of lipogenesis- and lipolysis-associated genes in LL and LKO mice. **(C)** Hepatic mRNA expression of lipogenesis- and lipolysis-associated genes in vehicle- and GW4064-treated LKO mice. **(D)** Representative immunoblots of hepatic ACC1, FASN, SCD1, ATGL, and HSL in vehicle- and GW4064-treated LKO mice. **(E)** Densitometric quantification of the immunoblots shown in **(D)**. **(F)** Relative mRNA expression of *Fxr* and *Shp* in primary LKO hepatocytes treated with GW4064. **(G)** mRNA expression of lipogenesis- and lipolysis-associated genes in GW4064-treated primary LKO hepatocytes. **(H)** Working model linking hepatic GHR deletion with reduced BSEP expression, altered bile acid compartmentalization, lower FXR/SHP signaling, changes in lipid metabolism-related proteins, and steatosis. Data are mean ± SEM. For **(B–C)**, n = 6 mice per group; for **(D–E)**, n = 4 mice per group; for **(F–G)**, n = 3 per group. *P < 0.05, **P < 0.01, and ***P < 0.001 by two-tailed unpaired Student’s t-test.

### GW4064 upregulates lipolytic genes in primary LKO hepatocytes

We next tested GW4064 directly in primary LKO hepatocytes to distinguish hepatocyte-intrinsic responses from systemic effects. Cells were treated with 1 μM GW4064 or vehicle for 24 h. GW4064 significantly increased *Fxr* and *Shp* mRNA expression, consistent with activation of FXR signaling in GHR-deficient hepatocytes (**Figure 5F**). GW4064 also reduced cellular lipid accumulation (**Supplementary Figure S3**) and significantly increased *Atgl* and *Hsl* expression without significantly altering *Acc1*, *Fasn*, or *Scd1* (**Figure 5G**). *Bsep* expression did not change significantly despite the induction of *Fxr* (**Supplementary Figure S2D**). These findings reproduce the hepatic transcriptional response to GW4064 in isolated LKO hepatocytes.

## Discussion

The association between MASLD and impaired GH action remains underrecognized (40, 41). In the present GEO dataset analysis, hepatic GHR or IGF1 expression was lower in MASLD and correlated positively with FXR expression. Although IGF1 expression is also influenced by nutritional and insulin status, systemic inflammation and hepatocellular function, hepatic IGF1 is a canonical transcriptional output of the GHR–JAK2–STAT5 axis and is widely used as a molecular indicator of hepatic GH action (42, 43). Disease severity and hepatocyte function may influence these expression patterns. Even so, the positive GHR/IGF1–FXR correlations are consistent with the lower FXR/SHP expression in LKO liver and support an association between hepatic GH signaling and FXR in MASLD.

Liver-specific *Ghr* knockout mice develop spontaneous steatosis and therefore provide a model in which to examine the role of liver GH signaling in MASLD progression (20). LKO livers accumulated bile acids, and bile acid abundance correlated positively with MASLD-related indices. Higher total and conjugated bile acids, including TαMCA, TβMCA, and TUDCA, coincided with lower FXR and SHP expression. TαMCA and TβMCA have been characterized as murine FXR antagonists (44). Although GUDCA was the principal bile acid implicated in the metformin response studied by Sun et al., that study also demonstrated that TUDCA inhibited FXR reporter activity and intestinal FXR signaling (45). Zhao et al. further showed that 50 μM TUDCA reduced FXR and SHP expression in L-02 hepatocytes (46). The altered bile acid pool may therefore contribute to reduced hepatic FXR signaling.

Hepatic bile acid abundance reflects the balance between synthesis and export. The accumulation of FXR-antagonistic non-12-OH bile acids did not appear to result from increased synthesis because *Cyp7a1* was unchanged and *Cyp7b1*, which contributes to non-12-OH bile acid synthesis, was downregulated. *Cyp7b1* is a GHR/STAT5-responsive hepatic gene, and liver STAT5 deletion has been shown to reduce male *Cyp7b1* expression (47). As the principal transporter for canalicular secretion of conjugated bile acids, BSEP plays a critical role in maintaining hepatic bile acid homeostasis. Experimental loss of BSEP disrupts bile acid metabolism and constrains biliary bile salt secretion (48, 49). This established role, together with the opposing changes in hepatic and gallbladder bile acids, supports reduced BSEP expression as a potential contributor to hepatic conjugated bile acid accumulation. BSEP is a direct transcriptional target of FXR, which activates its promoter through an IR-1 element (50). Reduced FXR signaling may therefore contribute to lower BSEP expression in LKO liver. However, GW4064 increased *Shp* expression without significantly changing *Bsep* expression in either LKO liver or primary LKO hepatocytes. This selective response suggests that GHR-associated signals influence *Bsep* expression, although *Bsep* induction may also require a different agonist dose or treatment duration.

Studies of congenital and adult-onset hepatic GHR disruption indicate that GH signaling influences several branches of hepatic lipid metabolism (18, 51). GW4064 improved steatosis in LKO mice, supporting a contribution of reduced FXR signaling to hepatic lipid accumulation in this model. FXR regulates hepatic lipid metabolism through several mechanisms, including inhibition of lipogenesis through the FXR–SHP–SREBP1c axis (52), an SHP/SREBP1c-independent pathway (53), and promotion of triglyceride hydrolysis and clearance (54). We found that the expression of genes involved in both lipogenesis and lipolysis was significantly altered. GW4064 increased ATGL and HSL expression without reversing the elevated expression of *Acc*, *Fasn*, or *Scd1*. This selective transcriptional response, together with reduced lipid accumulation in primary LKO hepatocytes, suggests that enhanced lipid mobilization contributes to the improvement in steatosis. GW4064 lowered serum TG in LKO mice, although baseline concentrations did not differ significantly between LL and LKO mice. Serum TG depends on the balance between triglyceride entry into and clearance from the circulation, rather than hepatic lipid content alone (52). Similar baseline concentrations are therefore compatible with a TG-lowering response to GW4064.

Clinical evidence supports the efficacy of GH therapy in improving liver lipid metabolism in MASLD and obesity (55, 56), yet safety concerns—including elevated cancer risk and glycemic dysregulation—remain (57, 58). These safety concerns motivate evaluation of downstream pathways, including FXR, as alternative intervention points in MASLD associated with GH deficiency. Liver-specific GHR ablation is a stringent model, but it isolates hepatic GH signaling mechanisms that may also be relevant when GH action is partially reduced in obesity and type 2 diabetes (13, 59). The human expression correlations provide clinical context for this association. Direct bile acid manipulation and BSEP rescue would help clarify how altered bile acid homeostasis contributes to reduced hepatic FXR signaling. Tissue-specific Fxr models and receptor blockade could then distinguish the hepatic FXR, intestinal FXR–FGF15 (53), and potential FXR-independent components of the response to GW4064. Studies using partial or adult-onset GH deficiency would help assess its relevance beyond complete hepatic GHR ablation. This study used only male mice. Hepatic GH–STAT5 signaling and its targets, including *Cyp7b1*, are sexually dimorphic (47), so studies in females are needed to assess whether the findings also apply to females.

In conclusion, hepatic GHR deletion alters bile acid composition, reduces BSEP and FXR/SHP expression, and promotes hepatic steatosis. GW4064 ameliorates steatosis and increases lipolysis-associated gene expression in LKO mice and primary LKO hepatocytes. In our proposed model, the enrichment of bile acids with reported FXR-antagonistic activity may link hepatic GHR loss to reduced FXR signaling (**Figure 5H**). These findings establish hepatic GHR as a regulator of bile acid and lipid homeostasis and support pharmacological FXR activation as a treatment approach for GHR-deficient steatosis.

## Data Availability

The previously published data reanalyzed in this study were obtained from the Gene Expression Omnibus (GEO) under accession numbers GSE135251 and GSE48452.

## Supplementary material

Supplementary Figure 1 Bootstrap assessment of WGCNA module stability. **(A)** SABRE H-indices for the blue, brown, and turquoise reference modules. **(B)** Absolute Pearson correlations between the eigengenes of each reference module and its best-matching reconstructed module, recalculated within each bootstrap dataset. Each point represents one bootstrap resampling. Boxes indicate the interquartile range, horizontal lines indicate medians, and whiskers extend to the most extreme observations within 1.5 times the interquartile range. Colors correspond to the reference module assignments. The module stability analyses were performed for networks constructed using metabolomics data from our previous study (22).

Supplementary Figure 2 *Bsep* expression and gallbladder responses to GW4064 treatment. **(A)** Hepatic *Bsep* mRNA expression. **(B)** Gallbladder weight. **(C)** Gallbladder-to-body-weight ratio. **(D)** *Bsep* mRNA expression in GW4064-treated primary LKO hepatocytes. Data are mean ± SEM. For **(A–C)**, n = 6 mice per group; for **(D)**, n = 3 per group. ns, not significant by two-tailed unpaired Student’s t-test.

Supplementary Figure 3 GW4064 treatment reduces lipid accumulation in primary hepatocytes isolated from LKO mice. **(A)** Representative bright-field and Oil Red O staining images. Scale bars, 50 μm. **(B)** Oil Red O-positive area expressed relative to the cell-covered area. Data are mean ± SEM. ***P* < 0.01.
