# Supplementary material for: Hepatic GHR deletion alters bile acid composition and links reduced FXR signaling to hepatic steatosis
Source: Front Endocrinol (Lausanne). 2026 Oct 15;17:1919362. doi: 10.3389/fendo.2026.1919362 (PMC13716597; doi:10.3389/fendo.2026.1919362)
Supplement: Supplementary file 1 [file Table1.docx]

**Supplementary Table S1. Primers used for real-time qPCR.**

| Target | Primer | Sequence (5’-3’) |
| --- | --- | --- |
| *Gapdh* | Gapdh-RT-F | GGGCTGGCATTGCTCTCAATG |
|  | Gapdh-RT-R | CATGTAGGCCATGAGGTCCAC |
| *Cyp7a1* | Cyp7a1-RT-F | AGCAACTAAACAACCTGCCAGTACTA |
|  | Cyp7a1-RT-R | GTCCGGATATTCAAGGATGCA |
| *Cyp7b1* | Cyp7b1-RT-F | TAGCCCTCTTTCCTCCACTCATA |
|  | Cyp7b1-RT-R | GAACCGATCGAACCTAAATTCCT |
| *Bsep* | Bsep-RT-F | CTGCCAAGGATGCTAATGCA |
|  | Bsep-RT-R | CGATGGCTACCCTTTGCTTCT |
| *Fxr* | Fxr-RT-F | TCCAGGGTTTCAGACACTGG |
|  | Fxr-RT-R | GCCGAACGAAGAAACATGG |
| *Shp* | Shp-RT-F | CGATCCTCTTCAACCCAGATG |
|  | Shp-RT-R | AGGGCTCCAAGACTTCACACA |
| *Atgl* | Atgl-RT-F | ATGTTCCCGAGGGAGACCAA |
|  | Atgl-RT-R | GAGGCTCCGTAGATGTGAGTG |
| *Hsl* | Hsl-RT-F | CCAGCCTGAGGGCTTACTG |
|  | Hsl-RT-R | CTCCATTGACTGTGACATCTCG |
| *Acc1* | Acc1-RT-F | GAATCTCCTGGTGACAATGCTTATT |
|  | Acc1-RT-R | GGTCTTGCTGAGTTGGGTTAGCT |
| *Fasn* | Fasn-RT-F | ATCGCCTATGGTTGTTGACC |
|  | Fasn-RT-R | TTCAGGGTCATCCTGTCTCC |
| *Scd1* | Scd1-RT-F | CCGGAGACCCCTTAGATCGA |
|  | Scd1-RT-R | TAGCCTGTAAAAGATTTCTGCAAACC |
